# Regulation of Cathepsin G Reduces the Activation of Proinsulin-Reactive T Cells from Type 1 Diabetes Patients

**DOI:** 10.1371/journal.pone.0022815

**Published:** 2011-08-05

**Authors:** Fang Zou, Nadja Schäfer, David Palesch, Ruth Brücken, Alexander Beck, Marcin Sienczyk, Hubert Kalbacher, ZiLin Sun, Bernhard O. Boehm, Timo Burster

**Affiliations:** 1 Division of Endocrinology and Diabetes, Center for Internal Medicine, University Medical Center Ulm, Ulm, Germany; 2 Panatecs, Tübingen, Germany; 3 Wroclaw University of Technology, Wroclaw, Poland; 4 Medical and Natural Sciences Research Center, University of Tübingen, Tübingen, Germany; 5 Institute of Diabetes, Zhongda Hospital Medical School, Southeast University, Nanjing, China; La Jolla Institute of Allergy and Immunology, United States of America

## Abstract

Autoantigenic peptides resulting from self-proteins such as proinsulin are important players in the development of type 1 diabetes mellitus (T1D). Self-proteins can be processed by cathepsins (Cats) within endocytic compartments and loaded to major histocompatibility complex (MHC) class II molecules for CD4^+^ T cell inspection. However, the processing and presentation of proinsulin by antigen-presenting cells (APC) in humans is only partially understood. Here we demonstrate that the processing of proinsulin by B cell or myeloid dendritic cell (mDC1)-derived lysosomal cathepsins resulted in several proinsulin-derived intermediates. These intermediates were similar to those obtained using purified CatG and, to a lesser extent, CatD, S, and V in vitro. Some of these intermediates polarized T cell activation in peripheral blood mononuclear cells (PBMC) from T1D patients indicative for naturally processed T cell epitopes. Furthermore, CatG activity was found to be elevated in PBMC from T1D patients and abrogation of CatG activity resulted in functional inhibition of proinsulin-reactive T cells. Our data suggested the notion that CatG plays a critical role in proinsulin processing and is important in the activation process of diabetogenic T cells.

## Introduction

Type 1 diabetes mellitus (T1D) is an organ/antigen-specific autoimmune disease manifested by infiltration of lymphocytes into pancreatic islets, resulting in insulitis and the destruction of β cells. Proinsulin is one of the major target autoantigens in T1D [1]. Consequently, processing and presentation of proinsulin exhibit a critical event in the disease pathology both in murine models such as non-obese diabetic mice and humans. The processing of proinsulin and identification of proinsulin-derived T cell epitopes can provide key elements of the disease process [2] and the alteration of the antigen processing machinery by the use of specific cathepsin inhibitors may represent a plausible strategy to interfere with ongoing autoimmune reaction [3].

Human antigen-presenting cells (APC) play an essential role in antigen-specific immunity and autoimmunity. Antigen processing within freshly isolated APC from human peripheral blood (primary APC) differs from that of B cell lines or *in vitro* generated monocyte-derived DC. The expression of the serine protease cathepsin G (CatG) has previously been demonstrated to be restricted mainly to primary APC compared to cell lines [4]. Therefore, the use of primary APC in assays addressing antigen processing is highly warranted [5], [6], [7].

Endocytic cysteine (CatB, C, F, H, L, S, V, X, and AEP), serine (CatG and CatA), and aspartic (CatD and CatE) cathepsins are active in processing of both antigens and autoantigens. Within the endocytic compartments, cathepsins truncate antigens into antigenic peptides which can subsequently be loaded onto major histocompatibility complex (MHC) class II molecules. The MHC/peptide complex is then transported to the cell surface where it is inspected by the T cell receptor of CD4^+^ T cells and initiates a specific response [8], [9], [10], [11], [12]. It was demonstrated by using CatS, B, and L deficient mice that these proteases are important in the onset of autoimmune diabetes [13], [14].

In this report, we show that CatG, D, S, and V is involved in proinsulin processing. Importantly, CatG is crucial in this process. The expression and activity of CatG are elevated in PBMC from T1D and is functionally controlled by a CatG inhibitor, suggesting relevance for potential immunotherapeutic approaches in humans.

## Results

### Cathepsin activity in PBMC from T1D vs. control donors

**Figure 1:**
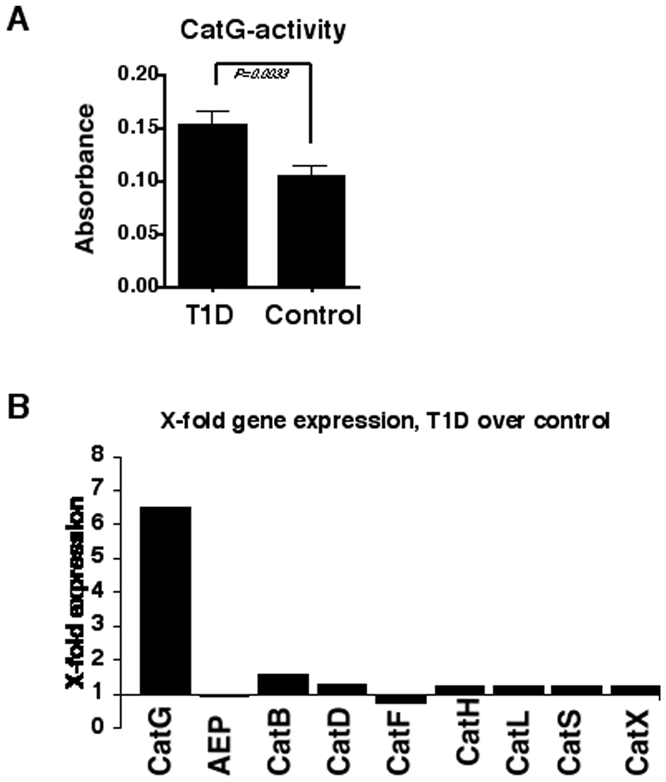
Expression of CatG in peripheral blood mononuclear cells (PBMC) from T1D patients vs. controls. A) CatG activity in PBMC was measured using the colorimetric substrate Suc-VPF-pNA. T1D, n = 25; controls, n = 29. The measurements were performed in duplicate. Statistical analysis was performed by using the unpaired, two-tailed Student's *t*-test. B) Gene expression was analyzed by quantitative RT-PCR in T1D (n = 5) and controls (n = 6). The multiple of a unit of gene expression over the control was set to 1.

Initially, we examined whether the protease activity might differ in PBMC from T1D patients compared to healthy control donors. PBMC-derived crude cell lysate was incubated with the colorimetric substrate Suc-VPF-pNA to quantify CatG activity between T1D and control donors. We found that CatG-activity was significantly elevated in T1D-derived PBMC (Fig. 1A). These findings were confirmed with the activity-based probe DAP [15] to visualize active CatG (Figure S1). Other classes of proteases associated with the antigen processing machinery, such as cysteine and aspartic cathepsins, were tested. Modestly reduced CatX activity was observed in some T1D donors while CatA, B, C, D, E, L, and AEP were found to be similar between T1D and controls (data not shown). Furthermore, we examined whether higher CatG activity in T1D was also due to higher CatG transcript levels. Therefore, PBMC from either T1D or control donors were tested for their relative cathepsin expression by performing quantitative RT-PCR. We found that CatG transcripts were elevated in samples from T1D patients, in contrast to other cathepsins (Fig. 1B). This demonstrates that both CatG transcript levels and activity are increased in T1D compared to healthy control donors.

### Regulation of cathepsins in PBMC after exposure to serum proteins

After determining higher CatG activity in PBMC from T1D patients, we further investigated CatG regulation in PBMC using serum samples from T1D donors. PBMC from control donors were isolated and incubated with serum proteins from T1D or control donors. None of these serum samples altered CatG activity or cysteine proteases in PBMC (data not shown), suggesting that serum factors from T1D patients were not responsible for increased CatG activity. Furthermore, we determined whether exposure to cytokines or toll-like receptor (TLR) ligands might provoke an increase of CatG activity in PBMC. TNF-α, IFN-γ, IL-1β, IL-17, TGF-β, and TLR1 to 9 ligands did not alter CatG activity in PBMC (data not shown). Thus, neither cytokines, serum samples from T1D patients, nor TLR ligands seem to be responsible for higher CatG activity found in PBMC from T1D patients.

### Downregulation of proteases within APC leads to reduced T cell activation

**Figure 2:**
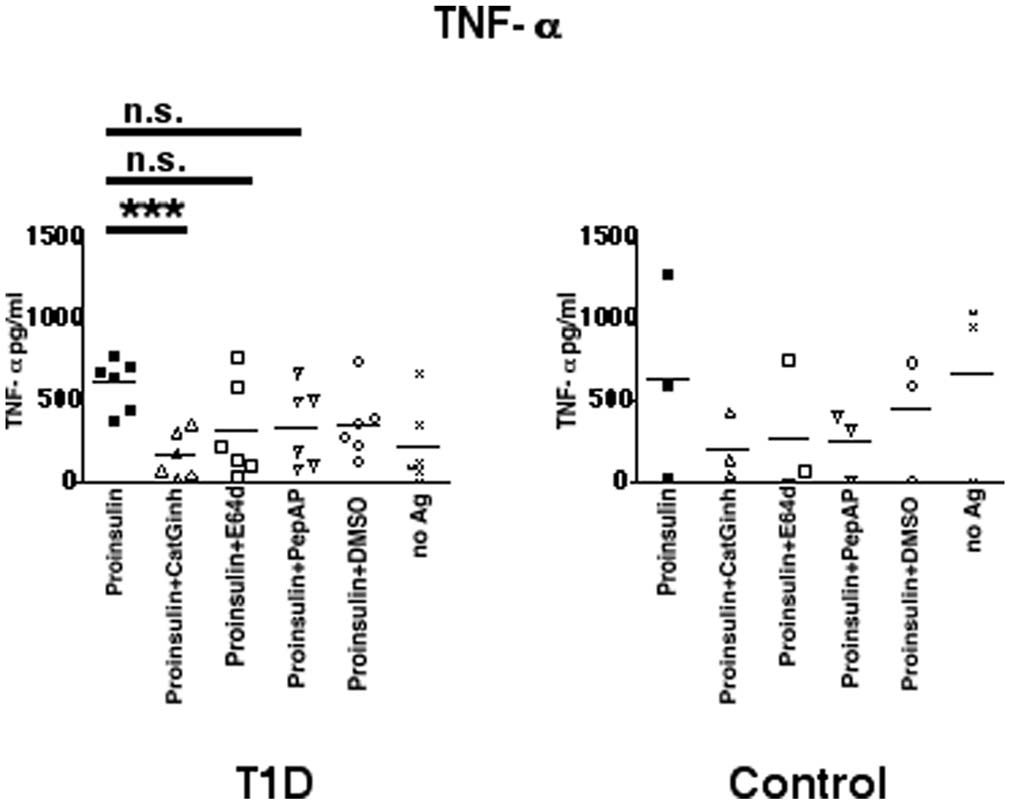
Regulation of proinsulin presentation after treatment with selected inhibitors. **P**BMC were incubated with CatG inhibitor (CatGinh.), E64d, or pepstatin A-penetratin (PepA-P) at 10 µM for five days. DMSO served as a vehicle control. TNF-α secretion was analyzed by ELISA and were carried out in quadruplicate. n = 6 T1D vs. n = 3 control donors. Statistical analysis was performed by using the unpaired, two-tailed Student's *t*-test. n.s., not significant and *** significant at p<0.001.

To determine which class of proteases is involved in proinsulin processing and presentation, cell permeable cathepsin inhibitors were co-incubated with proinsulin and PBMC from T1D or control donors in a functional T cell assay. The inhibitors used included a CatG inhibitor [16], pepstatin A-penetratin (PepA-P) to inhibit aspartic proteases [17], or E64d for cysteine protease inhibition [18]. PBMC pulsed with proinsulin induced T cell activation in autologous T cells from T1D analyzed by the detection of TNF-α (Fig. 2). In contrast, proinsulin did not activate T cells from control donors, indicated by the high TNF-α secretion in the no antigen (no Ag) sample. Co-treatment of PBMC with CatG inhibitor resulted in reduced frequencies of T cell stimulation in PBMC from T1D donors. In control donors levels of TNF-α were reduced, but did not reach significance. E64d, PepA-P, and DMSO did not significantly decrease TNF-α concentrations. Additionally, we analyzed the secretion of further proinflammatory cytokines, IFN-γ, IL-17, and IL-22, and found that CatG inhibitor downregulated these cytokines (Figure S2). E64d reduced only IL-17, PepA-P and DMSO had no effect. Thus, effective processing and presentation of proinsulin depend on the activity of CatG in T1D.

### Processing of proinsulin in vitro

**Figure 3:**
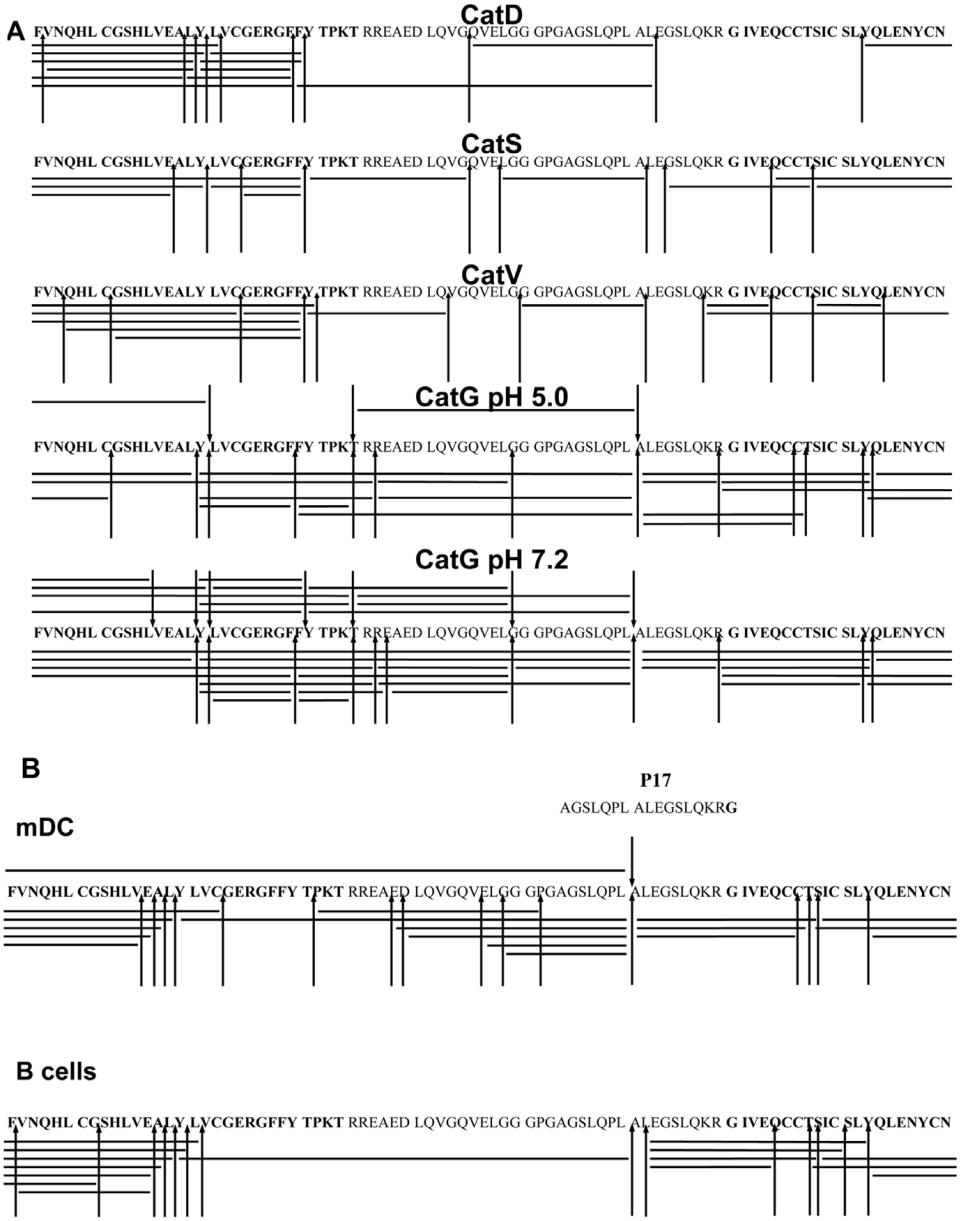
Processing of proinsulin by lysosomal proteases. **A**) Proinsulin was incubated with selected cathepsins *in vitro* for 2 h. The resulting fragments were analyzed by HPLC and mass spectrometry (LC-MS-MS and additionally MALDI-TOF). Bars indicate identified fragments and arrowheads designate cathepsin cleavage sites. CatV is expressed in cortical human thymic epithelial cells [39]. B) Proinsulin was incubated with B cell- or mDC1-derived lysosomal cathepsins and the resulting fragments were analyzed as described previously. Bars indicate identified fragments and arrowheads designate cathepsin cleavage sites.

Lang et al. described that porcine insulin-derived intermediates did not activate T cells when using a fixed B cell line as a source of APC. In contrast, a non-fixed B cell line was able to activate T cells, demonstrating that intermediates required further processing within the endocytic compartments in order to become functionally active [19]. Therefore, we mimicked the destination of non-reduced proinsulin within the antigen-processing compartment in an *in vitro* digestion experiment and incubated proinsulin with purified cathepsins, CatD, G, S, and V, which have endoprotease activity. The proteolytic degradation pattern was identified by mass spectrometry and the resulting fragments were summarized in a cathepsin cleavage site map (Fig. 3A). Intact proinsulin was successively degraded by all cathepsins used in the assay. CatD digested proinsulin between hydrophobic amino acid residues (F, L, and Y), while CatS and CatV cleavage occurred preferentially at branched hydrophobic amino acids in the P2 position (V and L). The tryptic, chymotryptic, Leu-ase, and Met-ase activity is restricted to human CatG compared to the chymotryptic activity of murine CatG [20], therefore human CatG cleaved proinsulin after positively charged and aromatic amino acids as well as after leucine. Processing of proinsulin was increased at neutral pH compared to acidic conditions. Having determined the digestion pattern using selected cathepsins, we incubated proinsulin with primary human B cell-or mDC1-derived lysosomal cathepsins and identified several cleavage sites. The majority of cleavage sites within proinsulin resembled those obtained with purified CatG (one key cleavage between LA at position C24/C25) and, to a lesser extent, by CatD, S, and V (Fig. 3B). Taken together, the processing of proinsulin by the combined action of proteases from mDC1 is controlled by CatG *in vitro*.

### T cells from T1D donors are activated by CatG-generated proinsulin peptides

**Figure 4:**
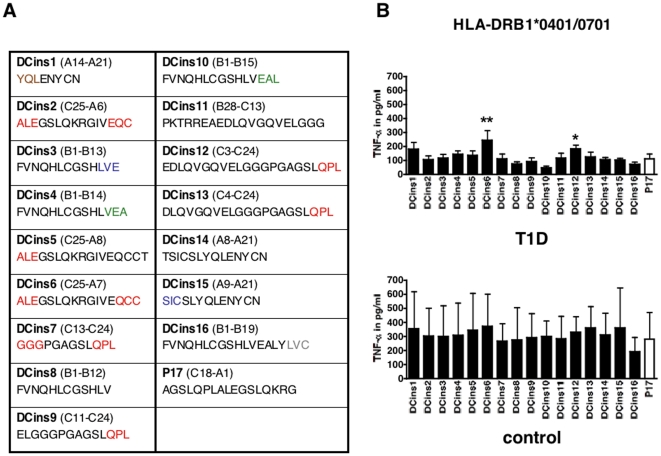
Functional T cell assay using human PBMC from T1D patients or control donors. A) Sequence of the resulting intermediates after digestion of proinsulin (DCins peptides): CatG (red), CatD (green), CatS (blue), CatV (grey), and CatG and CatD-derived fragments (brown). B) PBMC from T1D patients were incubated with DCins peptides or P17 (10 µg/ml) for five days. HLA-DRB1*0401/0701, n = 8 T1D donors, n = 2 control donors. The TNF-α secretion was monitored using ELISA. ELISA assays were completed in quadruplicate. n.s., not significant, * significant at p<0.05, and ** significant at p<0.01. Significance was determined by one-way ANOVA, post test Dunnett.

The peptides obtained after proteolytic degradation of proinsulin are summarized in Figure 4A. Strikingly, the generated fragment DCins10 was equal to a published peptide, which was protective in T1D and bound to DQB1*0602 [21]. Therefore, DCins10 was used as a “non-responder” and was compared to DCins peptides in a functional T cell assay. PBMC from T1D or control donors were incubated with DCins peptide 1–16 or P17, which was referred as a known T cell epitope [22], and the secretion of TNF-α was monitored by ELISA. We found levels of TNF-α secretion were significantly increased in response to DCins6 and DCins12 compared to DCins10 in HLA-DRB1*0401/0701 expressing T1D donors and none of these peptides tested elevated the secretion of TNF-α in control donors (Fig. 4B). Additional cytokines (IL-17 and IFN-γ) were tested. However, in several donors the level of cytokines was below the threshold of the assay and in the case of TGF-β1, no significant differences were found (data not shown). In addition, HLA-DRB1*0401/0301 T1D donors were tested. We did not observe substantial differences in the levels of TNF-α, with the exception of DCins14, incubated with the indicated DCins peptides (Figure S3). DCins6 as well as the C-terminal end of DCins12 and DCins13 were generated by CatG after the proteolytic digest of proinsulin, but only DCins6 and 12 elevated distinct T cell activation from HLA-DRB1*0401/0701 T1D donors.

### Vitamin D reduces CatG activity only in mDC1 from healthy donors

**Figure 5:**
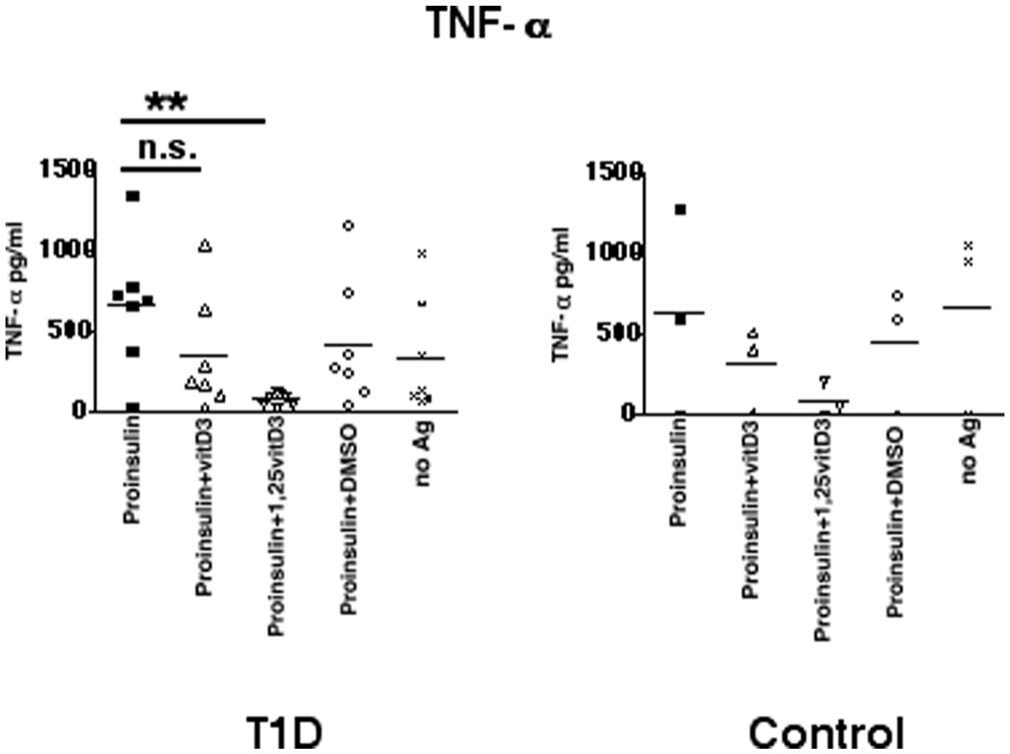
Regulation of proinsulin presentation by vitamin D. Functional T cell assay, PBMC from T1D (n = 6) or control donors (n = 3) were incubated with vitamin D_3_ (100 ng/ml) or 1α,25(OH)_2_D_3_ (100 ng/ml). TNF-α production was analyzed by ELISA in quadruplicate. Statistical analysis was performed by using the unpaired, two-tailed Student's *t*-test. n.s., not significant and ** significant at p<0.01.

To test our hypothesis that vitamin D might affect the antigen processing machinery in primary APC, PBMC from T1D or control donors were pulsed with proinsulin with or without vitamin D_3_ or 1α,25(OH)_2_D_3_. Proinsulin induced an adequate T cell response in PBMC from T1D patients. In contrast, addition of 1α,25(OH)_2_D_3_ reduced secretion of TNF-α (Fig. 5), IFN-γ, IL-17, and IL-22 (Figure S4). PBMC from control donors showed levels of cytokine production even with no antigen. We did not detect any differences of IL-6 or TGF-β1 (data not shown). These data show that vitamin D mitigates proinsulin-reactive T cell activation.

**Figure 6:**
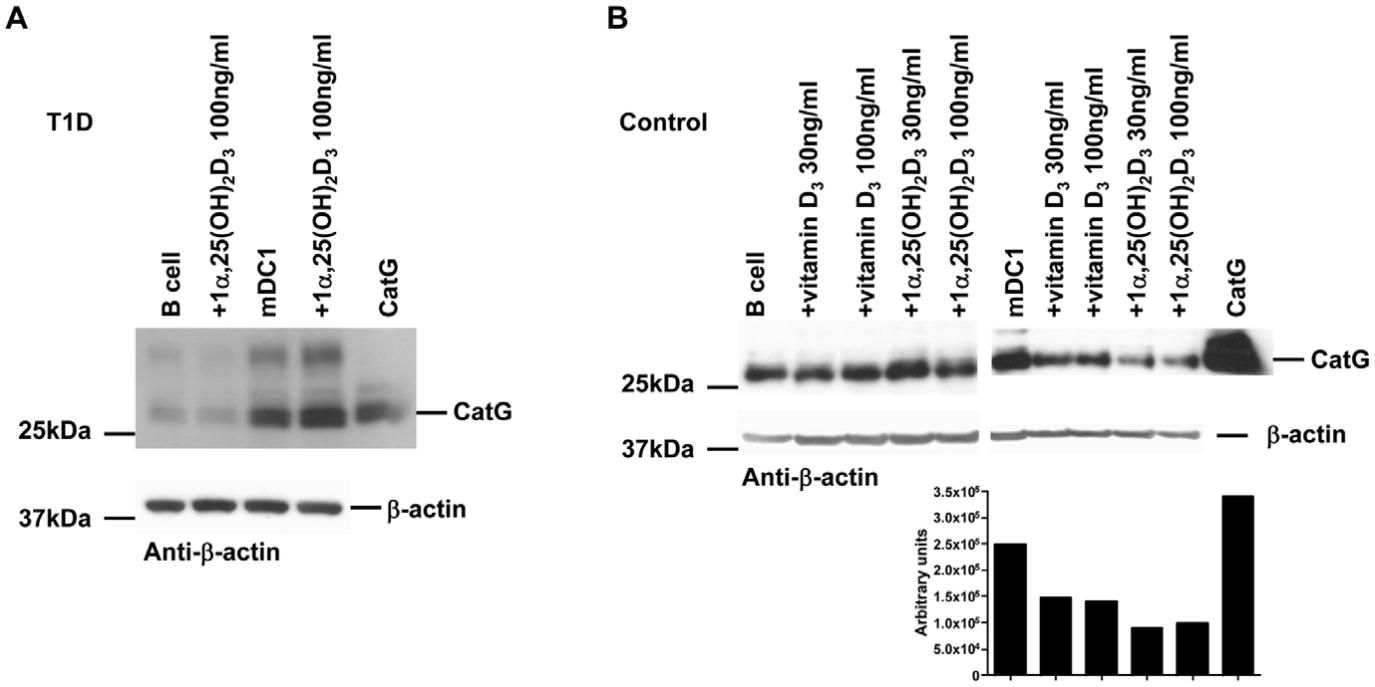
Regulation of CatG activity by vitamin D. B cells or mDC from (A) T1D or (B) healthy donors were co-cultured with vitamin D for 24 h. Equal amounts of cell lysate were incubated with the active site probe to visualize active CatG. Lower panel indicates quantification of band intensity. One representative active site label out of n = 4 T1D and n = 5 control donors is shown.

Next, we sought to determine whether the mitigation of T cell activation might be due to the activity of proteases located in the endocytic compartments of APC. B cells and mDC1 from T1D or control donors were incubated with physiological serum concentrations of prohormone cholecalciferol (vitamin D_3_) or the active form of vitamin D_3_, 1α,25-dihydroxyvitamin D_3_ (1α,25(OH)_2_D_3_). Subsequently the cathepsin activity was determined. We observed down-regulation of CatG activity in mDC1 from control donors when these cells were treated with 1α,25(OH)_2_D_3_, but not in T1D-derived mDC1 (Fig. 6A and B). Cysteine protease activity was not altered under the conditions used, in contrast to levels of MHC class II molecules were reduced after treatment with 1α,25(OH)_2_D_3_ only in control donors (Figure S5). Thus, mDC1 from T1D patients are resistant from CatG regulation by vitamin D.

## Discussion

Proinsulin is one of the major autoantigens in T1D. We found distinct fragmentation patterns when proinsulin was digested with primary human B cell- or mDC1-derived lysosomal cathepsins. DCins6 and DCins12 represented novel T cell epitopes in HLA-DRB1*0401/0701 T1D patients. Strikingly, CatG was identified to be the protease to control the processing of proinsulin due to the similar cleavage sites obtained by purified cathepsins compared to that acquired by mDC1-derived lysosomal cathepsins. Furthermore, CatG activity was higher in PBMC from T1D donors compared to controls and a CatG inhibitor reduced proinsulin-reactive T cell activation. Together, these results indicate a crucial role of CatG in proinsulin processing.

**Figure 7:**
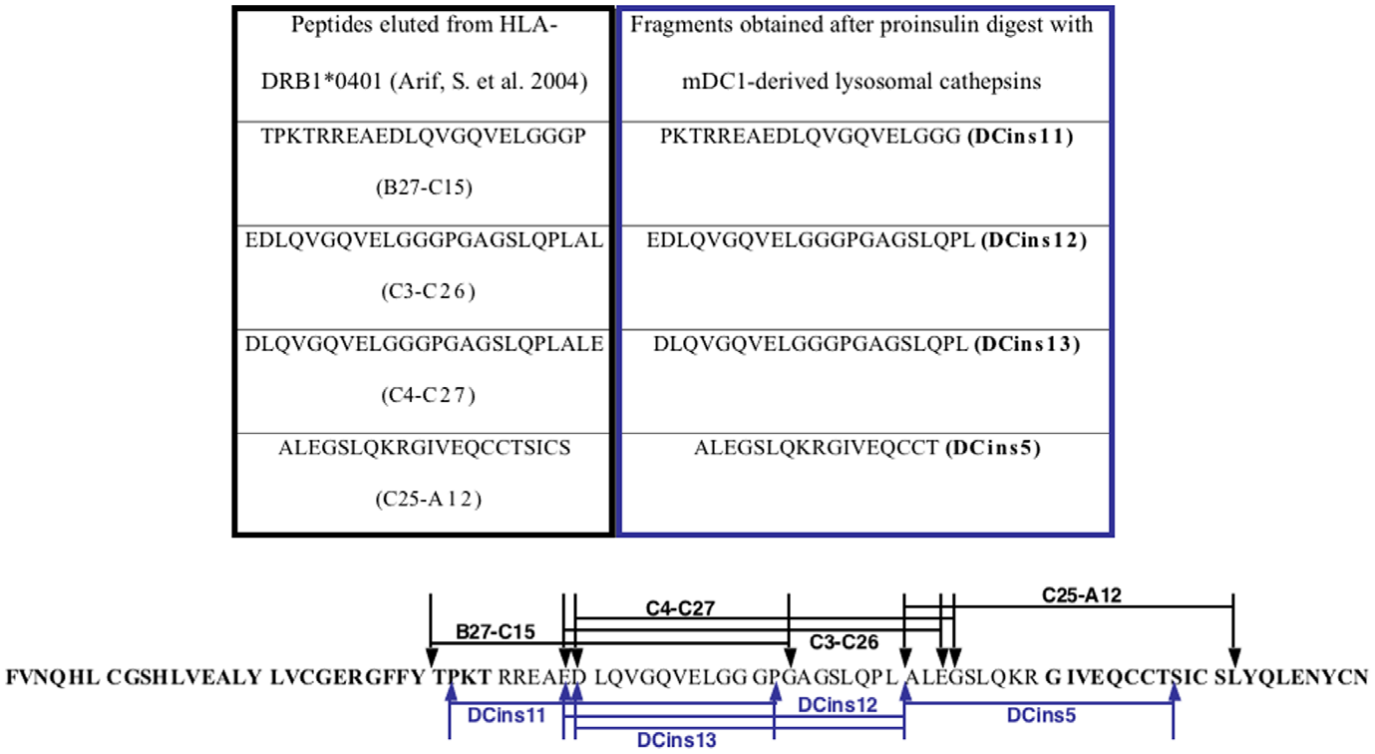
Sequence overview of identified peptides eluted from HLA-DRB1*0401/0401 BLC vs. peptides from proinsulin digested with mDC1-derived lysosomal proteases (DCins peptides).

Several T1D T cell epitopes were determined by overlapping peptide libraries or studies with a proinsulin pulsed B cell line, expressing HLA-DRB1*0401/0401, followed by MHC class II peptide elution [23]. Based on the results obtained herein unknown T cell epitopes were generated by endocytic proteases. Proinsulin processed by mDC1-derived cathepsins resulted in several fragments. DCins5, 11, and 12 were similar to B27-C15, C3–C26, and C25-A12 (Fig. 7), which were determined by peptide elution from proinsulin pulsed human BLC. Furthermore, the sequence of DCins10 was equal to the epitope (B1–B15) known to bind to the protective HLA allele HLA-DQB1*0602 [21]. In our experiments, this peptide did not induce the secretion of TNF-α, but did also bind to HLA-DRB1*0401 (Figure S6). Thus, we speculate that DCins10 (B1–B15) might be directly beneficial towards the suppression of autoaggressive T cells.

Two hypotheses exist for loading of antigens. First, antigens are processed by cathepsins into small antigenic peptides and can be loaded to MHC class II molecules. Second, a mechanism might occur in which initial processed antigens, resulting in larger intermediates, bind to MHC class II molecules to be further trimmed by exoproteases [24]. Intact insulin or proinsulin can not directly bind to MHC class II molecules. Therefore, insulin has to be internalized and processed to form a MHC class II-peptide complex [25]. We hypothesize that CatG, with its broad pH spectrum between pH 5–8, will degrade proinsulin after internalization into endocytic compartments and the resulting intermediates can subsequently bind to MHC class II molecules. Some of the bound peptides might be trimmed by exoproteases (CatA, B, C, H, and X) at either the N-or C-terminal end. This is supported by our findings where the *in vitro* generated peptides, DCins 6 and DCins12, activated proinsulin-reactive T cells, directly.

Inhibition of cysteine proteases, specifically CatS, reduces the processing of invariant chain and therefore the maturation of MHC class II molecules [26]. CatG and the aspartic protease CatD are not responsible for either invariant chain processing or maturation of MHC class II [26], [27], [28]. Thus, the inhibition of cysteine proteases can lead to both impaired MHC class II maturation and antigen processing, compared to CatG, where we provided direct evidence that CatG is a proper candidate in processing and presentation of proinsulin.

Low levels of vitamin D have been associated with autoimmune disorders including T1D [29]. It is known that vitamin D exhibits immunomodulatory capacity by abrogating the expression of MHC class II molecules in DC [30] and proinflammatory cytokines, particularly IFN-γ, IL-17, and IL-21, were reduced when treated with vitamin D [31]. Therefore, the therapeutic use of vitamin D might be a promising tool for the treatment of autoimmune diseases [29], [32]. Conversely, it has been shown that vitamin D has no major effect on residual pancreatic beta cell function [33]. CatG activity in mDC1 was decreased only in non-diabetic donors. Lower levels of CatG, found in control donors, might be explained by the presence of physiological levels of vitamin D. Alternatively, mDC1 from T1D were resistant to CatG regulation by vitamin D. This could explain higher levels of CatG found in T1D. However, there is no relation between CatG levels and reduced proinsulin presentation in a T cell assay when PBMC from T1D donors were incubated with vitamin D.

CatG is expressed in APC, granulocytes [4], and also in the lining layer cells of the synovial tissue. Consistently, CatG secretion has been shown to be induced at the inflammatory site of synovial fluid in rheumatoid arthritis (RA) patients [34]. Moreover, CatG deficient mice were less mortal than their wild type counterparts in a noninfectious mouse model of renal ischemia/reperfusion injury [35]. The combined inhibition of several serine proteases by the acute-phase serine protease inhibitor α1-antitrypsin (AAT) reduced the expression of proinflammatory cytokines and restored euglycemia in new onset diabetic NOD mice [36]. Here we demonstrated that the presentation of proinsulin-derived peptides to proinsulin-reactive T cells was significantly reduced by a CatG inhibitor. We described higher levels of CatG in PBMC from T1D patients compared to control donors. This result was not unexpected, since both T1D and RA are leukocyte-mediated diseases. In a clinical trial, oral administration of frankincense extract inhibited CatG activity in human blood indicating anti-inflammatory properties of this extract [37]. We speculate that the reduction of CatG activity by a natural substance without any side effects, for instance, oral intake/inhaling frankincense, might be beneficial to prevent T1D.

In summary, our data demonstrate that high levels of CatG activity found in T1D and the possibility to regulate CatG activity, might provide a basis for the modulation of autoaggressive T cells.

## Methods

### Peptide synthesis

Peptides were synthesized by the solid phase Fmoc strategy on a multiple peptide synthesizer Syro II (MultiSynTech, Witten, Germany). Peptides were purified by reversed-phase HPLC using a C18 column 125×8 mm (Grom, Herrenberg, Germany) and analyzed by mass spectrometry (Reflex IV, Bruker Daltonics, Bremen, Germany).

### PBMC from T1D and control donors

Peripheral blood mononuclear cells (PBMC) were Ficoll-isolated from heparinized blood and cryo-preserved in liquid nitrogen until used. T1D was diagnosed by the WHO guideline and informed written consent was obtained from each participant. Use of PBMC for *in vitro* studies was in accordance with IRB regulations. The ethics committee at the Ulm University, application No. 224/09 and 220/06, approved this study.

### T cell assay

1. T cell assay using inhibitors or vitamin D: proinsulin (10 µg/ml), CatG inhibitor I (10 µM, Calbiochem, Schwalbach, Germany), pepstatin A-penetratin (PepA-P, 10 µM, Kalbacher, MNF, University of Tübingen, Germany), E64d (10 µM, Enzo Life Sciences, Lörrach, Germany), vitamin D (100 ng/ml, cholecalciferol or 1α,25-dihydroxy vitamin D_3_; Sigma-Aldrich, Taufkirchen, Germany), or DMSO (0.1% final concentration, Sigma-Aldrich) were added to PBMC (1×10^6^ cells per well) and cultured for five days.

2. T cell assay using proinsulin-derived peptides (DCins peptides): DCins peptides or P17 (10 µg/ml, 6 µM) were added to PBMC (1×10^6^ cells per well). Cells were cultured for five days and supernatants were collected to determine cytokine production (TNF-α, IL-17, IL-6, IFN-γ, and TGF-β1) by ELISA (R&D Systems, Wiesbaden, Germany).

### Lysosomal proteases and in vitro processing

Lysosomal proteases were isolated from primary B cells and mDC1 by differential centrifugation. For *in vitro* processing, substrate solution (0.2 µg/µl peptide, 0.1 M sodium citrate pH 5.0, 2.5 mM DTT in a final volume of 50 µl) was incubated with lysosomal proteases (2.6 µg of total protein) at 37°C for up to 4 h. Peptides were incubated with CatG (Sigma-Aldrich), CatS, -L, -V, or -D (R&D Systems, Wiesbaden, Germany), in concentrations of 1–2 ng/µl for 2 h at 37°C.

### Identification of processing products

*In vitro* processed proinsulin samples were analyzed by on-line capillary LC/ESI-MS/MS. For LC separation, a gradient Agilent CapPump 1100 (Agilent, Waldbronn, Germany) connected to a 150×0.5 mm Zorbax SB-C18 5 µm capillary column (Agilent, Waldbronn, Germany) was used. The flow rate was 15 µl/min. Separation was performed using the following gradient: 0–5 min 5% system B, 5–45 min 5–80% system B, 45–50 min 80–95% system B, solvent A was 0.025% TFA in water (v/v) and solvent B was 0.024% TFA, 80% ACN in water (v/v). Before analysis, samples were reduced with dithiothreitol (DTT, 10 mM final concentration, 30 min incubation at 56°C) and subsequently applied (8 µl) to the column. UV chromatograms were acquired at 214 nm. Mass spectra were acquired in the positive ion mode using a HCT+ ion trap mass spectrometer (Bruker-Daltonics, Bremen, Germany) equipped with a standard ESI interface.

Electrospray voltage was set to 3850 V, dry gas (N2) to 6 l/min (at 325°C), the nebulizer to 15 psi. MS (300–2000 m/z), and MS/MS (200–3000 m/z, 1 V fragmentation amplitude) spectra were acquired at a scan speed of 26,000 m/z/sec. The data-dependent MS/MS analyses included the acquisition of a survey spectrum (m/z 300–2000) followed by MS/MS spectra (m/z 200–3000) of the two most abundant ions in the survey scan (1 min active exclusion). Un-interpreted MS/MS data were automatically analyzed using BioTools 2.2 and SequenceEditor 2.2 (Bruker-Daltonics). Assignment of fragment-ions was confirmed by manual comparison of acquired signals with predicted fragment-ions generated by the MS-Product component of ProteinProspector (http://prospector.ucsf.edu/). In addition, the fragments were analyzed by MALDI-TOF (Reflex IV, Bruker Daltonics, Bremen, Germany).

### Active site label and Western blot

Human peripheral blood mononuclear cells (PBMC) were freshly isolated from buffy coats of healthy blood donors or T1D donors by density gradient centrifugation. Myeloid dendritic cells (mDC1, CD1c^+^) and B cells (CD19^+^) were positively selected using the appropriate magnetic cell separation kit (Miltenyi Biotec, Bergisch Gladbach, Germany) following the manufacturer's protocol. For the activity-based label and Western blot, freshly isolated cells were treated with vitamin D (30 ng/ml or 100 ng/ml, vitamin D_3_ (cholecalciferol) or 1α,25-dihydroxy vitamin D_3_; Sigma-Aldrich, Taufkirchen, Germany) for 24 h at 37°C.

Cells were lysed (10 mM Tris [pH 7.5], 150 mM NaCl, and 0.5% NP-40), adjusted for equal amounts of total protein (quantified by the Bradford assay). Cell lysate was added to PBS (pH 7.4) in the presence of active site label to visualize active serine proteases and incubated for 1 h at room temperature. Samples were resolved by 12% sodium dodecyl sulfate polyacrylamide gel electrophoresis (SDS-PAGE), transferred to a polyvinylidenfluorid (PVDF) membrane, and visualized using streptavidin-horseradish peroxidase (HRP, Vectastain, Burlingame, CA, USA). For Western blot analysis, 20 µg of protein from crude cell extracts were subjected to SDS-PAGE, and the immunoblot was performed using anti-HLA-DR antibody (CHAMP, L. Stern, University of Massachusetts, MA, USA). Anti-β-actin antibody and secondary HRP-conjugated antibodies were obtained from Sigma-Aldrich (Taufkirchen, Germany). The use of human cells for in vitro studies was in accordance with IRB regulations. The ethics committee at the Ulm University, application No. 224/09 and 220/06, approved this study.

### Determination of CatG activity

Kinetic measurement of CatG activity was accomplished by adding 15 µg of PBMC-derived cell lysate from the indicated samples to the colorimetric substrate Suc-Val-Pro-Phe-pNA (200 µM) in buffer (0.5 M MgCl_2_ in PBS, pH 7.4) as previously described in [38]. The enzyme assays were performed in duplicate at 37°C, and absorption was determined at 405 nm (absorbance microplate reader, EL808, BioTek, Winooski, VT, USA).

### Quantitative PCR

Total RNA was extracted from freshly isolated PBMC (8×10^6^ cells) from T1D or control donors using the RNeasy Mini Kit (Qiagen, Hilden, Germany). Genomic DNA was removed with DNase (Qiagen). Reverse transcription of 740 ng of total RNA was performed using the RT2 First Strand Kit (SABioscience, Frederick, MD, USA) according to the manufacturer's instructions.

Gene specific primer pairs were selected with the Husar Genius software (DKFZ, Heidelberg, Germany) to span exon-intron junctions and generate an amplicon of 150 bp in length. Quantitative reverse transcriptase polymerase chain reaction (qRT-PCR) was accomplished using RT2 SYBR Green qPCR Master Mix (SABioscience). The qRT-PCR gene expression was detected with the ABI 7500 Fast Real Time PCR System and the appropriate System Sequence Detection Software (Applied Biosystems, Carlsbad, CA, USA). 18S rRNA was used as an endogenous control to normalize levels of cDNA between preparations.

Data were collected and analyzed as follows: In total 11 donors, T1D (n = 5) and control donors (n = 6) were analyzed for their relative cathepsin expression. The data were analyzed with the ΔΔCt method as recommended by the supplier (SABiosciences). Delta cycles (ΔΔCt) were determined by subtracting the value of threshold Ct housekeeping gene from the Ct value of the gene of interest. The mean values of T1D or control were determined and included in the formula, ΔΔCt = ΔCt T1D-ΔCt control. The x-fold change of cathepsin expression of T1D over control was analyzed by adding ΔΔCt values to 2^(−ΔΔCt)^.

The following oligonucleotide primers (Thermo Fisher Scientific, Ulm, Germany) were used:

18S rRNA forward primer, 5′- CGGCTACCACATCCAAGGAA-3′ and reverse primer, 5′-GCTGGAATTACCGCGGCT-3′; human AEP GAAGC CTGTGAGTCTGGGTC, CAGTCCCCCAGGTACGTG; CatB CTGTG TAT TCGGACTTCCTGC, CCAGGAGTTGGCAACCAG; CatD AACT GCTGGACATCGCTTG, AGGTACCCGGAGAGGCTG; CatF ATAT GAGTCAAAGGAAGAAGCCC, GATCAC TGAACTTGGTGACTCC; CatG CCCCTACATGGCGTATCTTCA, TTGCTTCCC CAGCAATGAG; CatH ACTGGCTGTTGGGTATGGAG, AGGCCACACATGTTCTTTCC; CatL ACCAAGTGGAAGGCGATG, TTCCCTTCCCTGTATTCCTG; CatS ACTCA GAATGTGAATCATGGTG, TTCTTGCCATC CGAATA TATCC; CatX GGGAGGGAGA AGATGATGG, ATGTGGTGTC CTGGTATTCG.

### Statistical analysis

Data depict means ± standard error of the mean (S.E.M.). Statistical analysis was performed using one-way ANOVA (post test, Dunnett) or the unpaired, two-tailed Student's *t*-test (GraphPad Prism 3 software). A value of p<0.05 was considered significant.

## Supporting Information

Figure S1 **Crude PBMC lysate from T1D or control donors were incubated with the serine activity-based probe DAP22c.** This inhibitor detects active CatG by forming a covalent bond to the active center of the protease. Since DAP contains biotin, protease activity can be revealed *via* streptavidin-HRP detection. CatG-activity was significantly elevated in T1D-derived PBMC. Representative sample from n = 32 T1D and n = 36 control donors analyzed with DAP22c is shown (n = 9 T1D, n = 7 control were titrated).
(TIF)

Figure S2 **Regulation of proinsulin presentation.** PBMC were incubated with CatG inhibitor (CatGinh.), E64d, or pepstatin A-penetratin (PepA-P) at 10 µM for five days. DMSO served as a vehicle control. Cytokine secretion was analyzed by ELISA in quadruplicate. n = 6 T1D vs. n = 3 control donors. Statistical analysis was performed by using the unpaired, two-tailed Student's *t*-test. n.s., not significant and * significant at p<0.05.
(TIF)

Figure S3 T cell assay, PBMC from T1D donors, HLA-DRB1*0401/0301, n = 5, n = 3 control donors, were cultured with DCins peptides for five days at 37°C. Secretion of TNF-α was determined by ELISA. ELISA assays were done in quadruplicate. n.s., not significant and * significant at p<0.05.
(TIF)

Figure S4 **Regulation of proinsulin presentation by vitamin D. PBMC from T1D (n = 6) or control donors (n = 3) were incubated with vitamin D_3_ (100 ng/ml) or 1α,25(OH)_2_D_3_ (100 ng/ml).** Cytokine production was analyzed by ELISA in quadruplicate. Statistical analysis was performed by using the unpaired, two-tailed Student's *t*-test. n.s., not significant, * significant at p<0.05, and ** significant at p<0.01.
(TIF)

Figure S5 **5 µg of cell lysate were incubated with reaction buffer (0.1 M citrate, pH 5.0 and 50 mM DTT) in the presence of DCG-04 (10 µM; probe kindly donated by M. Bogyo, Stanford University, Palo Alto, CA, USA) to visualize active CatX, B, H, and S (left panel).** Immunoblot to visualize both α-and β-chain of MHC class II (CHAMP antibody), control donors (right panel, n = 3 donors), and T1D (left, lower panel, n = 3 donors). Quantification of band intensity, right lower panel.
(TIF)

Figure S6 Peptide binding assay. Fluorescent labeled hemagglutinin-peptide (HA-AMCA) was preloaded to one of the high-risk T1D HLA-DR alleles, HLA-DRB1*0401 followed by the addition of indicated peptides. Binding or competition were analyzed by high performance size exclusion chromatography (HPSEC). Peptide DCins12 bound to HLA-DRB1*0401 with the same capacity as P17. In contrast, DCins1, 3, 4, 5, 6, 7, 9, 10, 11, 13, and 16 bound with modest affinity. No binding was observed with DCins8, 14, or 15. Notably, the sequence of DCins10 is equal to the peptide B1–B15, which binds to HLA-DQB1*0602 and is protective to T1D.
(TIF)
