# Supplementary figures and images for: Regulation of Cathepsin G Reduces the Activation of Proinsulin-Reactive T Cells from Type 1 Diabetes Patients
Source: PLoS One. 2011 Aug 5;6(8):e22815. doi: 10.1371/journal.pone.0022815 (PMC3151250; doi:10.1371/journal.pone.0022815)

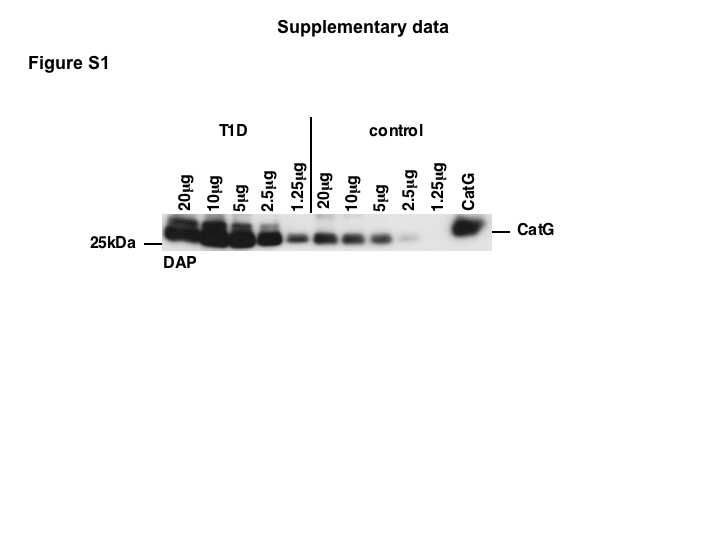

Supplement: Figure S1 — Crude PBMC lysate from T1D or control donors were incubated with the serine activity-based probe DAP22c. This inhibitor detects active CatG by forming a covalent bond to the active center of the protease. Since DAP contains biotin, protease activity can be revealed via streptavidin-HRP detection. CatG-activity was significantly elevated in T1D-derived PBMC. Representative sample from n = 32 T1D and n = 36 control donors analyzed with DAP22c is shown (n = 9 T1D, n = 7 control were titrated). (TIF) [file pone.0022815.s001.tif]

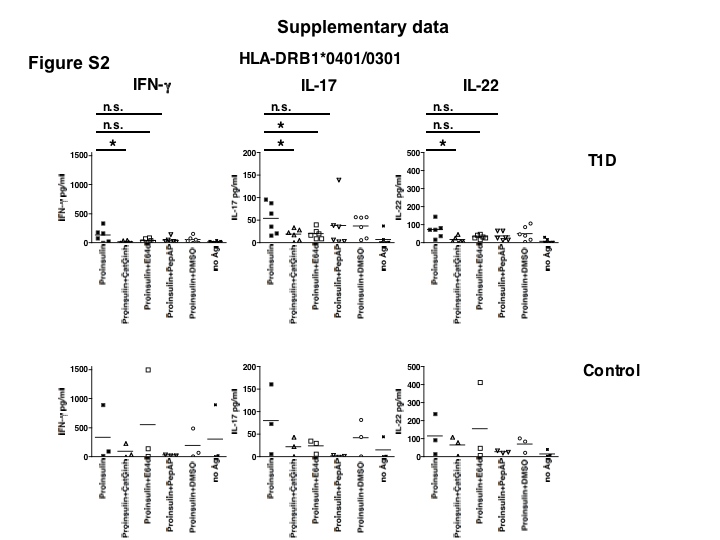

Supplement: Figure S2 — Regulation of proinsulin presentation. PBMC were incubated with CatG inhibitor (CatGinh.), E64d, or pepstatin A-penetratin (PepA-P) at 10 µM for five days. DMSO served as a vehicle control. Cytokine secretion was analyzed by ELISA in quadruplicate. n = 6 T1D vs. n = 3 control donors. Statistical analysis was performed by using the unpaired, two-tailed Student's t-test. n.s., not significant and * significant at p<0.05. (TIF) [file pone.0022815.s002.tif]

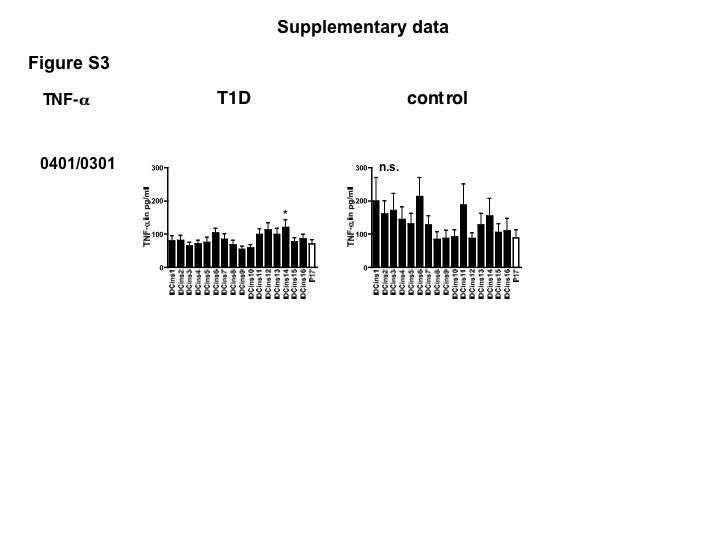

Supplement: Figure S3 — T cell assay, PBMC from T1D donors, HLA-DRB1*0401/0301, n = 5, n = 3 control donors, were cultured with DCins peptides for five days at 37°C. Secretion of TNF-α was determined by ELISA. ELISA assays were done in quadruplicate. n.s., not significant and * significant at p<0.05. (TIF) [file pone.0022815.s003.tif]

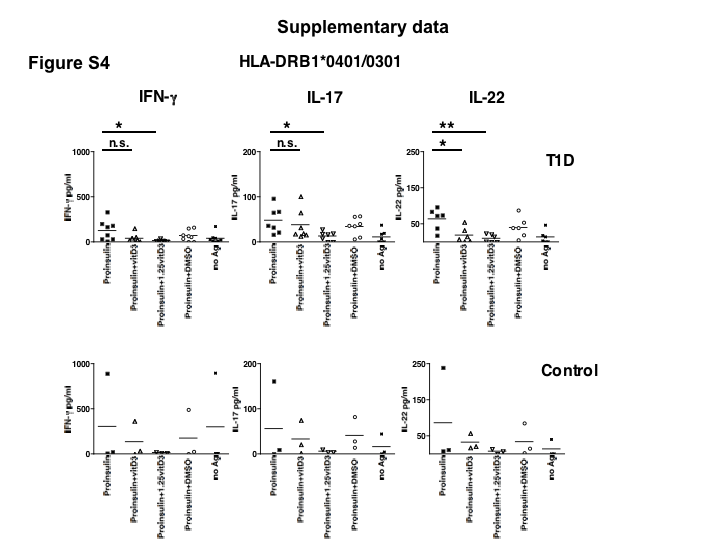

Supplement: Figure S4 — Regulation of proinsulin presentation by vitamin D. PBMC from T1D (n = 6) or control donors (n = 3) were incubated with vitamin D3 (100 ng/ml) or 1α,25(OH)2D3 (100 ng/ml). Cytokine production was analyzed by ELISA in quadruplicate. Statistical analysis was performed by using the unpaired, two-tailed Student's t-test. n.s., not significant, * significant at p<0.05, and ** significant at p<0.01. (TIF) [file pone.0022815.s004.tif]

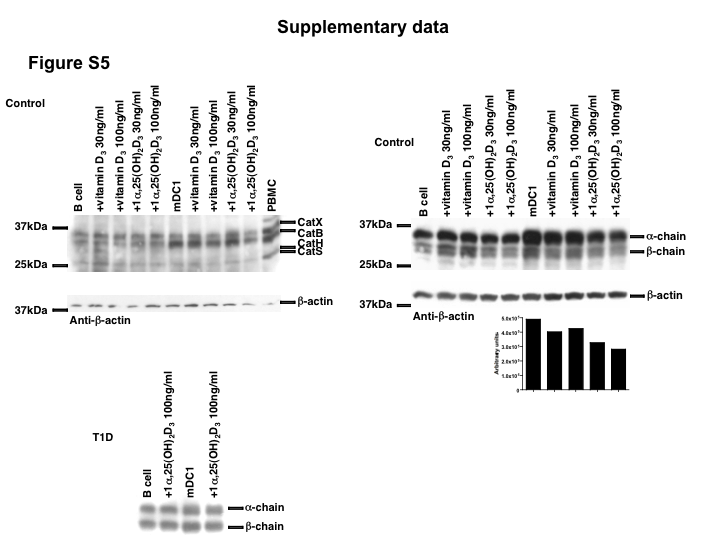

Supplement: Figure S5 — 5 µg of cell lysate were incubated with reaction buffer (0.1 M citrate, pH 5.0 and 50 mM DTT) in the presence of DCG-04 (10 µM; probe kindly donated by M. Bogyo, Stanford University, Palo Alto, CA, USA) to visualize active CatX, B, H, and S (left panel). Immunoblot to visualize both α-and β-chain of MHC class II (CHAMP antibody), control donors (right panel, n = 3 donors), and T1D (left, lower panel, n = 3 donors). Quantification of band intensity, right lower panel. (TIF) [file pone.0022815.s005.tif]

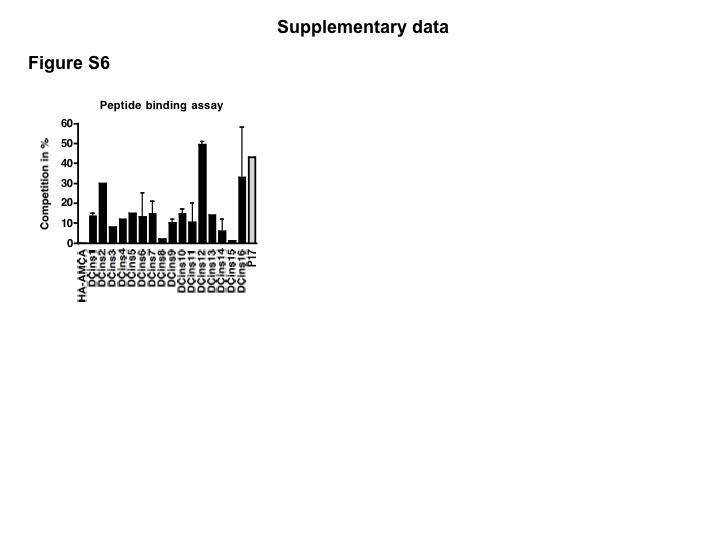

Supplement: Figure S6 — Peptide binding assay. Fluorescent labeled hemagglutinin-peptide (HA-AMCA) was preloaded to one of the high-risk T1D HLA-DR alleles, HLA-DRB1*0401 followed by the addition of indicated peptides. Binding or competition were analyzed by high performance size exclusion chromatography (HPSEC). Peptide DCins12 bound to HLA-DRB1*0401 with the same capacity as P17. In contrast, DCins1, 3, 4, 5, 6, 7, 9, 10, 11, 13, and 16 bound with modest affinity. No binding was observed with DCins8, 14, or 15. Notably, the sequence of DCins10 is equal to the peptide B1–B15, which binds to HLA-DQB1*0602 and is protective to T1D. (TIF) [file pone.0022815.s006.tif]
